# Proportion of vitamin D deficiency in children/adolescents with type 1 diabetes: a systematic review and meta-analysis

**DOI:** 10.1186/s12887-024-04683-5

**Published:** 2024-03-16

**Authors:** Xin Yang, Min Chai, Meng Lin

**Affiliations:** 1 Department of Pediatric Genetics, Metabolism and Endocrinology Nursing, West China Second University Hospital, Sichuan University, No. 1416, Section 1, Chenglong Avenue, Chengdu, Sichuan China; 2 Key Laboratory of Birth Defects and Related Diseases of Women and Children, Sichuan University, Ministry of Education, No. 1416, Section 1, Chenglong Avenue, Chengdu, Sichuan China

**Keywords:** Epidemiology, Type 1 diabetes, Vitamin D, Children/adolescents

## Abstract

**Background:**

The impact of vitamin D on type 1 diabetes has been a controversial topic in public health. Furthermore, significant differences in the proportion of vitamin D have been noted. The purpose of this systematic review was to determine the overall proportion of vitamin D deficiency in children/adolescents with type 1 diabetes (T1D).

**Methods:**

Based on six electronic databases (PubMed, Web of Science, Embase, Ovid Medline, ProQuest, and Cochrane Library), eligible studies since the databases’ inception up to April 2022 were searched. Reference lists were also manually searched to identify additional studies. Overall, studies with statistical information on vitamin D deficiency in children and adolescents with T1D were included, and a random effects model was applied for the meta-analysis. In addition, subgroup and sensitivity analyses were carried out to evaluate heterogeneity, and publication bias was evaluated by using Egger’s test.

**Results:**

A total of 45 studies involving 6,995 participants met the inclusion criteria; these included 25 countries covering Africa, Oceania, Europe, North America and Asia. The proportion of vitamin D deficiency in children/adolescents with T1D was 45% (95% confidence interval [CI] 37–54%, *I*^2^ = 97.94%). Subgroup analysis further revealed that the publication year, study design, vitamin D classification, season and geographical region significantly contributed to the variation in the reported incidence of vitamin D deficiency.

**Conclusions:**

The results of the meta-analysis showed that the proportion of vitamin D deficiency among T1D children/adolescents was 45%. In addition, the proportion remains higher, which has important implications for adapting health and social care systems.

**Supplementary Information:**

The online version contains supplementary material available at 10.1186/s12887-024-04683-5.

## Background

Type 1 diabetes (T1D), an autoimmune disease that affects pancreatic beta cells, is one of the most common endocrine disorders affecting children and young adults worldwide [1–3]. According to statistics, 2.15 out of every 1,000 people that are 19 years or younger and from only 6 regions of the United States were diagnosed with T1D in 2017 [4]. Furthermore, a pooled analysis conducted in 26 European centers revealed a yearly increase of 3.4% in the incidence rate of T1D [5]. It is also referred to as a chronic autoimmune disease, and there is not current medical technology for its cure. This condition inflicts substantial lifetime morbidity, affecting patients both during their childhood and throughout their adult lives [6]. Therefore, we must determine an effective management strategy for children and adolescents with type 1 diabetes and their families. However, diabetic ketoacidosis (DKA) has a high incidence of recurrence and is a leading cause of mortality among patients with T1D, resulting in an elevated burden for patients, families, hospitals, and healthcare providers [7]. Therefore, it is important to find ways to prevent the prevalence of T1D. In this context, one potential factor, vitamin D (VD), has attracted the attention of many scholars. Indeed, vitamin D deficiency/insufficiency represents a substantial but modifiable public health risk that deserves increased attention [8], as the number of T1D patients suffering from vitamin D deficiency has been increasing rapidly [9].

Vitamin D deficiency seems to be a common issue even in the general population. Measurement of the circulating form of vitamin D that best describes total body stores, namely, 25-hydroxyvitamin D, can be unreliable despite the many sophisticated methodologies that have been proposed and implemented [10]. Similarly, evidence from clinical studies showing a beneficial role of vitamin D in different disease states has been controversial and at times speculative [11]. Additionally, significant differences in the proportion of vitamin D have been noted.

Vitamin D deficiency has been shown to be common in children/adolescents with T1D [12]. Vitamin D, also called calciferol, is an essential fat-soluble vitamin that plays a considerable role in the growth and strength of bones by controlling calcium and phosphorus homeostasis [13]. In addition to its role in calcium homeostasis, it has an antiproliferative and immunosuppressive properties that regulate cell proliferation and differentiation [14, 15]. According to a review, vitamin D deficiency can potentially influence the incidence, comorbidity, and progression of T1D. Furthermore, in a cross-sectional study, 70% of children with T1D were reported to be vitamin D deficient [16].

However, epidemiological data based on various studies have shown that the prevalence of vitamin D deficiency among individuals with T1D varies greatly between 4% and 92% [17, 18], indicating inconsistency and uncertainty in the currently available information.

Several factors could explain the above variations in the prevalence of vitamin D deficiency between the different sources of data. First, different criteria are used to assess vitamin D deficiency. In addition, the quality and number of examined studies as well as the sampling procedures used in recorded studies tend to be heterogeneous, thereby leading to variable and possibly imprecise estimates. These methodological challenges highlight the importance of assessing the prevalence of vitamin D deficiency in children/adolescents with T1D through a systematic approach.

Although different reviews on the subject are already available, to our knowledge, no systematic reviews and meta-analyses have been conducted to reliably establish the proportion of vitamin D deficiency in children/adolescents with T1D. Therefore, by synthesizing information from different sources, the current systematic review not only sought to address the above knowledge gap but also to evaluate how the characteristics of studies influence estimations of the prevalence of diabetes.

## Methods

### Protocol and registration

This study was performed according to the Preferred Reporting Items for Systematic Reviews and Meta-Analyses (PRISMA) checklist [19]. The protocol was registered in the International Prospective Register of Systematic Reviews (CRD 42,022,301,690). This study did not include human research; therefore, no ethics approval was sought.

### Search strategy

A thorough literature search was carried out to find published articles on the proportion of vitamin D deficiency in children and/or adolescents with T1D. Studies published from the inception of the database up to the end of April 2022 were considered. The following electronic databases were used for the search: PubMed, Web of Science, Cochrane Library, Ovid Medline, Embase and ProQuest. The following key terms were used: ‘diabetes mellitus insulin dependent’ or ‘diabetes mellitus juvenile onset’ or ‘juvenile onset diabetes mellitus’ or ‘IDDM’ or ‘diabetes juvenile onset’ or ‘diabetes mellitus sudden onset’ or ‘type 1 diabetes mellitus’ or ‘diabetes autoimmune’ or ‘diabetes mellitus brittle’ or ‘Ketosis-Prone’ or ‘ketosis prone diabetes mellitus’ or ‘Adolescen*’ or ‘Teen*’ or ‘Youth*’ or ‘Child*’ or ‘Vitamin D’ and Medical Subject Headings (MeSH) terms ‘diabetes mellitus, type 1’, ‘diabetes mellitus’, ‘Adolescent’, ‘Child’ and ‘Vitamin D’. The research team then created a search strategy based on the MeSH terms and free-text phrases. In this case, the team browsed through the references listed in the published research to discover additional potentially suitable studies, with no restrictions regarding the date or language of publication. The search strategies are shown in Appendix S1.

### Study selection and eligibility criteria

The following materials were selected: (1) observational studies (cross-sectional designs, longitudinal research baseline cross-sectional data, cohort studies, and case–control studies); (2) participants/subjects included children/adolescents (under 20 years of age) with T1D; (3) the proportion of vitamin D deficiency in children and/or adolescents with T1D was described in peer-reviewed literature; and (4) the primary outcome measured the proportion of vitamin D deficiency in children and/or adolescents with T1D while vitamin D insufficiency and vitamin D sufficiency were secondary outcome indicators. Studies were excluded if they were commentaries, reviews, posters, case reports or letters to the editor; if clear data were not provided; or if the article reported duplicated data.

### Data extraction

Two independent reviewers (XY and MC) examined the publications’ titles and abstracts, followed by their entire texts to ensure that they met the inclusion criteria. Any discrepancies were settled through communication with a third reviewer (ML). Two separate researchers retrieved information from the selected papers, including the first author’s name, year, title, country, study design, and sample size and characteristics (sex, age, diagnostic criteria for diabetes, classification of vitamin D, etc.).

### Quality assessment

The methodological quality of the included studies was independently evaluated by different reviewers (XY and MC) using appropriate instruments. The Newcastle–Ottawa Scale (NOS) [20] was used to assess the quality of the cohort and case–control studies. In this case, the NOS scores ranged from 0 to 9, with studies with NOS scores greater than 6 considered of reasonably high quality, scores 5–6 considered of medium quality and scores less than 5 deemed to be of low quality. In addition, using the “star system,” each included study was evaluated in three domains: representativeness of the study group during selection, group comparability and exposure or outcome ascertainment. Finally, the Agency for Healthcare Research and Quality (AHRQ) methodology checklist was used to measure the validity of the cross-sectional studies. Each study was evaluated based on 11 items from the checklist [21], with the quality rated as follows: decent quality = 8–11, moderate quality = 4–7, and poor quality = 0–3. If no agreement could be reached, a third researcher (ML) was recruited to settle the dispute.

### Statistical analysis

The data analysis was carried out using the meta-analysis function in STATA software (Stata version 12.0; StataCorp, College Station, TX, USA). For the evaluation of the pooled effect, a 95% confidence interval (CI) was used, and *P* < 0.05 indicated statistical significance. Random effects were used to pool studies reporting the proportion of vitamin D deficiency in children and/or adolescents with T1D. The *I*^2^ index was subsequently used to examine between-study heterogeneity. If the *I*^2^ value was less than 50%, a nonsubstantial level of heterogeneity was assumed and the meta-analysis applied a fixed effects model. Conversely, an *I*^2^ value greater than 50% was indicative of substantial heterogeneity, for which a random effects model was used. The impact of a single study on the overall estimate of proportion was also investigated by eliminating each study in turn during a sensitivity analysis. Additionally, when there was more than one study in a subgroup, subgroup analyses were performed based on overall study design, vitamin D classification, season (winter, summer, spring, and fall) and geographical location (Asia, Europe, Oceania, Africa, North America, and South America). Funnel plots and Egger’s test results were eventually combined to explore potential publication bias in this meta-analysis. The trim and fill method, developed by Duval and Tweedie, is employed to identify and correct funnel plot asymmetry potentially induced by publication bias. The presence of publication bias in the study findings was assessed using the nonparametric trim and fill method.

## Results

### Search results and study characteristics

**Fig. 1:**
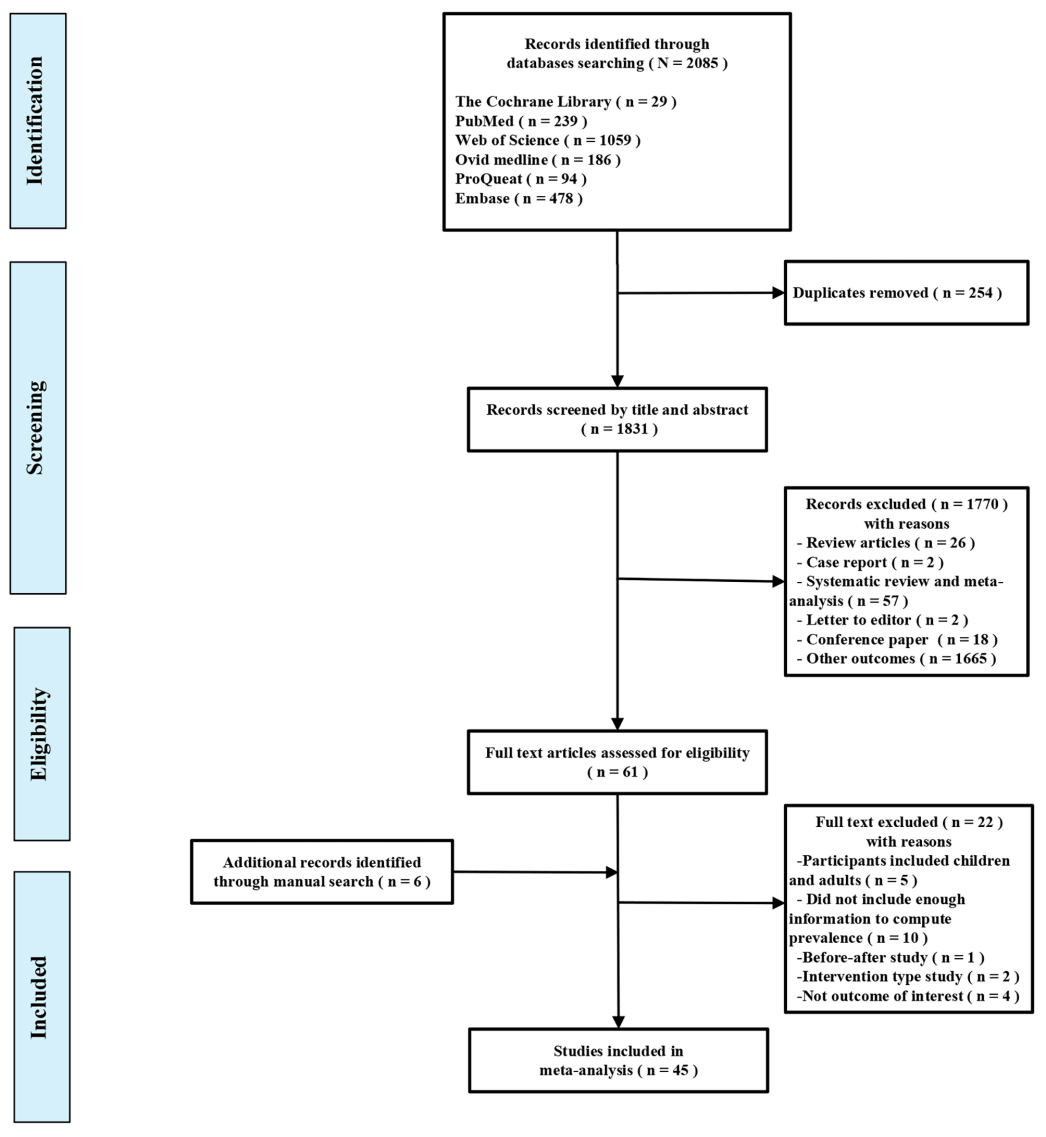
Flow diagram of the identification of eligible studies

A total of 2,085 titles and abstracts were retrieved from the electronic database searches, and after removing 254 duplicates, 1,831 were screened based on their titles and abstracts. This process yielded 61 full-text studies that were subsequently evaluated for eligibility. Six supplementary articles were also found to be eligible from the reference lists of the included studies. After reviewing the full texts, 45 studies were ultimately included in the meta-analysis. A summary of the selection process for the studies is presented in Fig. 1.

### Descriptions of the included studies

Out of the 45 studies, 19 had cross-sectional designs [16, 22–39], 23 had case–control studies [40–62], 2 had baseline cross-sectional data from a longitudinal study [63, 64] and one had baseline data from a cohort study [65]. The reported data also included 6,995 participants, mostly aged ≤ 18 years, 2,436 of whom were children/adolescents with T1D and vitamin D deficiency (sample size *n* = 13 ~ 1,426). Overall, T1D cases were mainly ascertained on the basis of criteria established by the World Health Organization (WHO) and the American Diabetes Association and the European Diabetes (EURODIAB) collaboration, while levels of 25-hydroxyvitamin D (25(OH)D) were measured using a radioimmunoassay kit or high-performance liquid chromatography (HPLC). Similarly, vitamin D status was ascertained mainly on the basis of the Endocrine Society Clinical Practice Guideline, the Institute of Medicine guidelines, the Australian Consensus Statement Criteria and the Central European Guidelines. Among the countries included in the studies, seven were conducted in America, four were conducted in Turkey, three were carried out each in Korea, Iran and India, two each were conducted in Australia, the United Kingdom, Egypt, Spain, Italy and the Kingdom of Saudi Arabia, and one was performed in China, Indonesia, Poland, Kuwait, Canada, Bangladesh, Slovakia, Switzerland, Boston, Ukraine, Tunisia, Iraq and Germany. The main characteristics of the 45 included studies are shown in Table 1. In accordance with the recommended NOS and AHRQ criteria, only studies of acceptable quality were included in the present meta-analysis; eight studies received 9 stars [50, 52, 55–57, 61, 62, 65], ten studies received 8 stars [42, 47–49, 51, 53, 54, 58–60], five studies received 7 stars [41, 43–46], and one study received 6 stars [40]. When using the quality assessment criteria from the AHRQ, three studies received a score of 11 [24, 28, 64], ten received a score of 10 [16, 22, 27, 28, 30, 32, 33, 35, 38, 39], three received a score of 9 [23, 31, 34], one received a score of 8 [26], one received a score of 7 [36] and two received a score of 5 [25, 37]; the quality assessment is shown in Appendix S2. Therefore, no article from the meta-analysis was excluded for quality reasons.

**Table 1:** Characteristics of the selected studies

Study	Year	Design	Country	Sample	Age (years)	Gender (M/F)	Diabetes duration (year)	Definition of diabetes mellitus	Vitamin D measure	Definition of vitamin D	25(OH)D (ng/ml)	Vitamin D cut-off	Vitamin D deficiency *n*(%)	NOS/ AHRQ
Bener	2009	Case-control	UK	170	< 16	88/82	NR	venous blood glucose values equal or > 6.7mmol/L	RIA	NR	15.8 ± 9.2	①	154 (90.6)	9
Borkar	2010	Case-control	Indian	50	6 ~ 12	29/21	NR	ADA	HPLC	NR	20.02 ± 10.63	②	29 (58.0)	9
Daga	2012	Case-control	Indian	13	< 18	6/7	NR	NR	RIA	NR	11.36 ± 4.74	②	12(92.3)	8
Azab	2013	Case-control	Egypt	80	6 ~ 16	34/46	17 (3 ~ 52) (m)	WHO criteria	ELISA	AAP	24.7 ± 5.6	③	44 (55)	9
Lieberman	2013	Case-control	USA	211	12 ~ 19	51/160	10.9 ± 3.2	Islet cell antibody	Clinical lab	IMG	27.7 ± 0.7	②	47 (22)	9
Greer	2013	Case-control	Australia	56	NR	28/28	NR	NR	Clinical lab	ACSC	31.53 (28.77–34.29)	③	5 (8.9)	8
Franchi	2013	Case-control	Italy	58	1.1 ~ 16	32/26	NR	ADA	Chemiluminescent assay	NR	NR	②	39 (67.2)	8
Jung	2014	Case-control	Korea	102	< 18	41/61	NR	NR	Chemiluminescent assay	NR	14.5 ± 6.4	②	77 (75.4)	8
Wierzbicka	2016	Case-control	Poland	60	< 18	28/32	5.1 ± 3.9	NR	ECLIA	ESCPG	15.3 ± 7.0	②	49 (81.7)	8
Rasoul	2016	Case-control	Kuwait	216	< 15	104/112	NR	ISPAD/WHO	EIA	ESCPG	13.84 ± 6.66	②	182 (84.3)	9
Kim	2017	Case-control	Korea	42	9 ~ 14	12/30	6.4 ± 3.0	NR	^125^I-labeled radioimmunoassay	ESCPG	20.0 ± 6.4	③	24 (57.1)	8
Ziaei-Kajbaf	2018	Case-control	Iran	85	1 ~ 15	40/45	NR	NR	ELISA	NR	5.13 ± 4.24	②	65 (76.5)	7
Liu	2018	Case-control	China	296	8.99 ± 3.75	147/149	NR	NR	Non-radioactive EIA	the Global Consensus ^1^	19.51 ± 6.11	④	39 (13.2)	7
Federico	2018	Case-control	Italy	82	2 ~ 18	44/38	9.4 ± 3.9	NR	HPLC	ESCPG	21.79 ± 10.94	②	41 (50.0)	7
Bae	2018	Case-control	Korea	85	6 ~ 20	37/48	NR	NR	RIA	ESCPG	21.6 ± 8.5	②	41 (48.2)	8
Sonia	2016	Case-control	Tunisia	29	12 ~ 18	14/15	35.03 ± 42.4(m)	ADSC	RIA	NR	17.4 ± 1.0	②	15 (51.7)	8
Mansi	2021	Case-control	Iraq	104	< 5	36/68	NR	NR	NR	NR	NR	⑨	83 (79.8)	7
Soliman	2015	Case-control	Egypt	53	6 ~ 18	27/26	NR	ADA	Immun-diagnostik EIA	NR	7.65 ± 2.52	⑤	45 (84.9)	9
Rochmah	2022	Case-control	Indonesia	31	< 18	18/13	1.0 (0 ~ 11)	ISPAD	ELFA	ESCPG	26.11 (13.95–52.11)	②	4 (12.9)	8
Setty-Shah	2014	Case-control	USA	22	2 ~ 13	12/22	NR	ADA	Chemiluminescent assay	AAP and IMG	24.44 ± 6.04	③	3 (13.6)	9
Ghandchi	2012	Case-control	Iran	60	5 ~ 25	32/28	NR	NR	HPLC	NR	NR	⑤	51 (85.0)	8
Biliaieva	2022	Case-control	Ukraine	94	10 ~ 18	NR	NR	NR	Electrochemiluminescence	EPGC and IMG	NR	②	64 (68.1)	6
Polat	2022	Case-control	Turkey	29	9 ~ 16	NR	NR	NR	NR	ESCPG	NR	①	16 (55.2)	7
Raab	2014	Cohort	Germany	244	3 ~ 9	132/112	NR	ADA	RIA	NR	19.91 ± 0.60	②	125 (51.2)	9
Janner	2010	Cross-sectional	Switzerland	129	NR	69/60	NR	ADA	Chemiluminescent assay	NR	18.31 (16.51–20.15)	②	78 (60.5)	10
Svoren	2009	Cross-sectional	Boston	128	< 18	69/59	4.1 ± 5.6	NR	RIA	NR	26.8 ± 6.7	②	19 (15.0)	10
Mutlu	2011	Cross-sectional	Turkey	120	3 ~ 20	65/55	3.2 ± 2.3	NR	ELISA	AAP	25.6 ± 16.2	⑦	27 (22.5)	10
Thnc	2011	Cross-sectional	Turkey	100	< 20	NR	56.4 ± 3.7 (m)	NR	HPLC	NR	NR	⑤	28 (28.0)	7
Vojtkova	2012	Cross-sectional	Slovakia	58	9 ~ 19	30/28	NR	ADA	Biochemical	NR	NR	⑥	21 (36.2)	10
Ataie-Jafari	2012	Cross-sectional	Iran	53	8 ~ 18	14/39	13.2 ± 6.1	NR	RIA	NR	NR	②	41 (77.0)	9
The	2013	Longitudinal	USA	1426	< 20	730/696	10.2 ± 3.9 (m)	NR	Chemiluminescent assay	IMG	NR	④	300 (21.0)	11
Savastio	2016	Longitudinal	Italy	64	< 12	NR	5.6 ± 3.9	ADA	Chemiluminescent assay	ESCPG	17.71 ± 9.62	⑥	41 (64.0)	10
Al-Zubeidi	2016	Cross-sectional	USA	185	< 19	81/94	NR	NR	Chemiluminescent assay	ESCPG	NR	⑥	33 (17.8)	10
Al	2016	Cross-sectional	Kingdom of Saudi Arabia	301	1 ~ 18	140/161	7.7 ± 3.7	ADA	Chemiluminescent assay	PES	14.06 ± 6.37	⑦	103 (34.2)	11
Zambrana-Calví	2016	Cross-sectional	Spain	90	< 18	46/44	NR	ISPAD	HPLC	NR	NR	⑦	12 (13.3)	10
Al Sawah	2016	Cross-sectional	USA	197	7 ~ 18	85/112	NR	NR	(LC-MS/MS)	NR	21.88 ± 7.13	②	80 (40.6)	9
Giri	2017	Cross-sectional	UK	271	7.7 ± 4.4	NR	NR	NR	Tandem Mass Spectrometry	the Global Consensus^1^	12.90 ± 3.29	④	40 (14.8)	8
ALkharashi	2019	Cross-sectional	Kingdom of Saudi Arabia	100	2 ~ 12	50/50	NR	NR	Biochemical	NR	14.06 ± 0.56	③	70 (70.0)	10
Zabeen	2021	Cross-sectional	Bangladesh	60	11 ~ 15	18/42	NR	ISPAD	RIA	NR	12.97 (9.3–18.0)	⑦	31 (51.7)	10
Segovia-Ortí	2020	Cross-sectional	Spain	67	0 ~ 14	31/36	NR	ISPAD	Chemiluminescent assay	ESCPG	NR	②	13 (19.4)	9
Carakushansky	2020	Cross-sectional	USA	395	3 ~ 18	202/193	NR	NR	(LC-MS/MS)	NR	NR	⑧	17 (4.7)	11
KOR	2018	Cross-sectional	Turkey	106	2 ~ 18	44/65	4.46 + 2.8	ISPAD	Chemiluminescent assay	NR	27.11 ± 14.33	④	7 (6.6)	5
Yeshayahu	2012	Cross-sectional	Canada	271	12 ~ 18	138/133	7.2 ± 3.6	NR	(LC-MS/MS)	NR	NR	⑧	89 (32.8)	10
Saki	2017	Cross-sectional	India	85	8 ~ 18	39/46	4.4 ± 2.8	Two positive autoantibody tests	HPLC	ESCPG	18.0 ± 12.2	②	52 (61.2)	10
Kaur	2011	Cross-sectional	Australia	517	8 ~ 20	NR	7.2 ± 3.5	NR	LIAISON	ANZ	28.09 ± 9.21	③	80 (15.5)	5

**†** NR, not reported; ACSC, Australian consensus statement criteria; LC-MS/MS, liquid chromatography-mass spectrometry; RIA, radioimmunoassay; ADAC, American Diabetes Association criteria; ECLIA, Electrochemilumines-cence immunoassay; EIA, enzyme immunoassay; ADSC, American diabetes society criteria; ELISA, ELISA assay kit; ADA, American Diabetes Association; AAP, The American Academy of Pediatrics; HPLC, high-performance liquid chromatography ESCPG, Endocrine Society clinical practices Guideline; IMG, the Institute of Medicine guidelines; EPGC, the Endocrine Practice Guidelines Committee; ELFA, enzyme immunoassay with enzyme-linked fluorescence assay;PES, the Drug and Therapeutics Committee of the Lawson Wilkins Pediatric Endocrine Society; ANZ, Australia and New Zealand: a consensus statement; 1the Global Consensus, the Global Consensus Recommendations on Prevention and management of Nutritional Rickets ;①vitamin D deficiency < 30ng/mL;vitamin D sufficiency = 30 ~ 80ng/mL; ②vitamin D deficiency < 20ng/mL; vitamin D insufficiency = 20 ~ 30ng/mL; VDS>30ng/L; ③vitamin D deficiency < 20ng/mL;vitamin D sufficiency>20ng/L; ④vitamin D deficiency < 12ng/ml; vitamin D insufficiency = 12 ~ 20ng/ml; vitamin D sufficiency ≥ 20ng/ml; ⑤vitamin D deficiency < 10ng/mL; vitamin D insufficiency = 10 ~ 20ng/mL; vitamin D sufficiency ≥ 20ng/mL; ⑥vitamin D deficiency = 10 ~ 20ng/mL; vitamin D insufficiency = 20 ~ 30ng/mL; vitamin D sufficiency ≥ 30ng/mL; ⑦vitamin D deficiency ≤ 15ng/mL;vitamin D insufficiency = 15 ~ 20ng/mL;vitamin D sufficiency ≥ 20ng/ml; ⑧vitamin D deficiency ≤ 15ng/mL;vitamin D insufficiency = 15 ~ 29ng/mL;vitamin D sufficiency ≥ 30ng/ml; ⑨vitamin D deficiency < 25ng/mL; vitamin D sufficiency ≥ 25ng/mL; ng/ml*2.496 = nmmol/L

### Meta-analyses and data synthesis

**Fig. 2:**
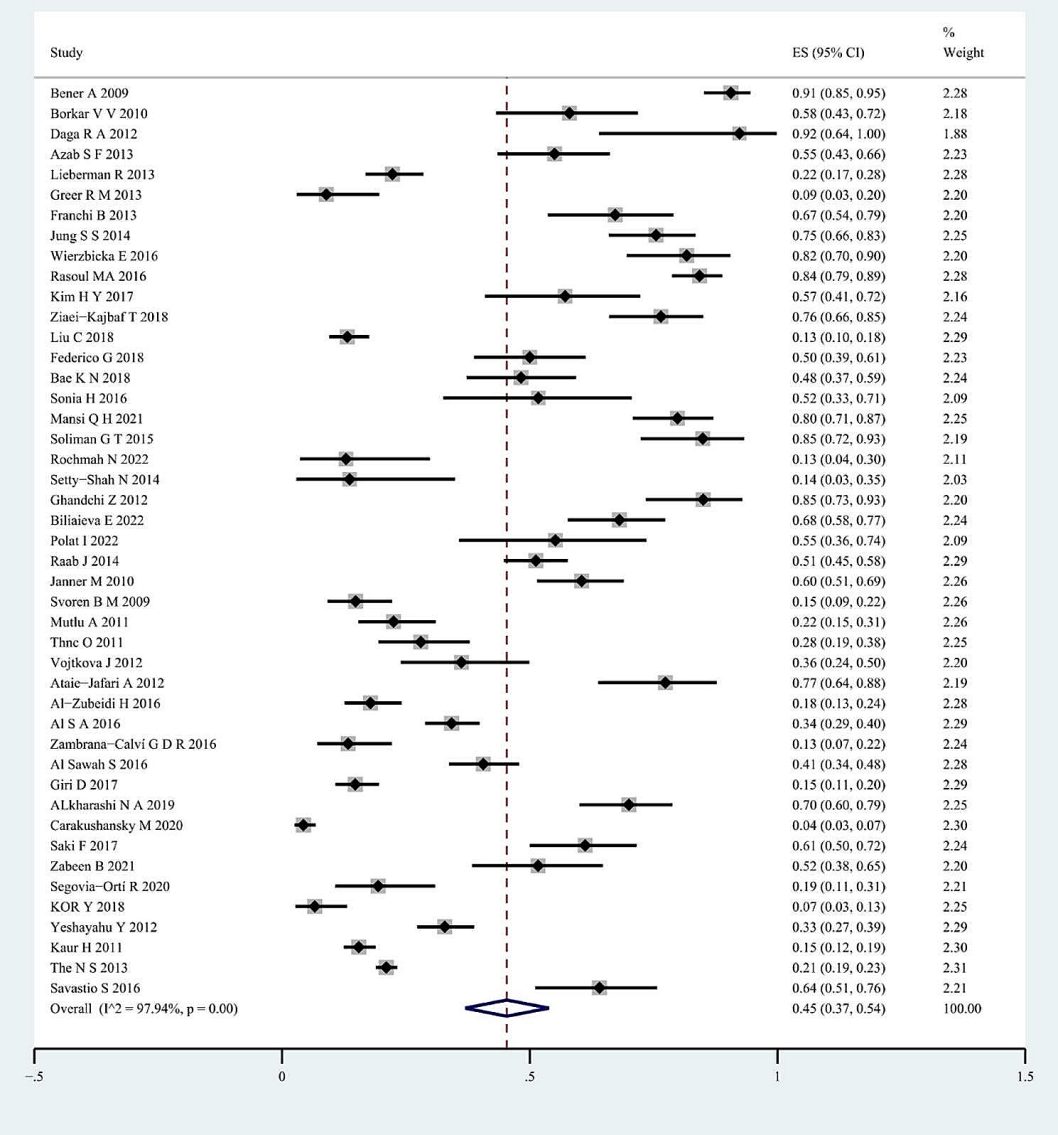
Forest plots for the total proportion of vitamin D deficiency in children/adolescents with type 1 diabetes. The diamond represents the pooled odds ratio and 95% confidence interval

**Fig. 3:**
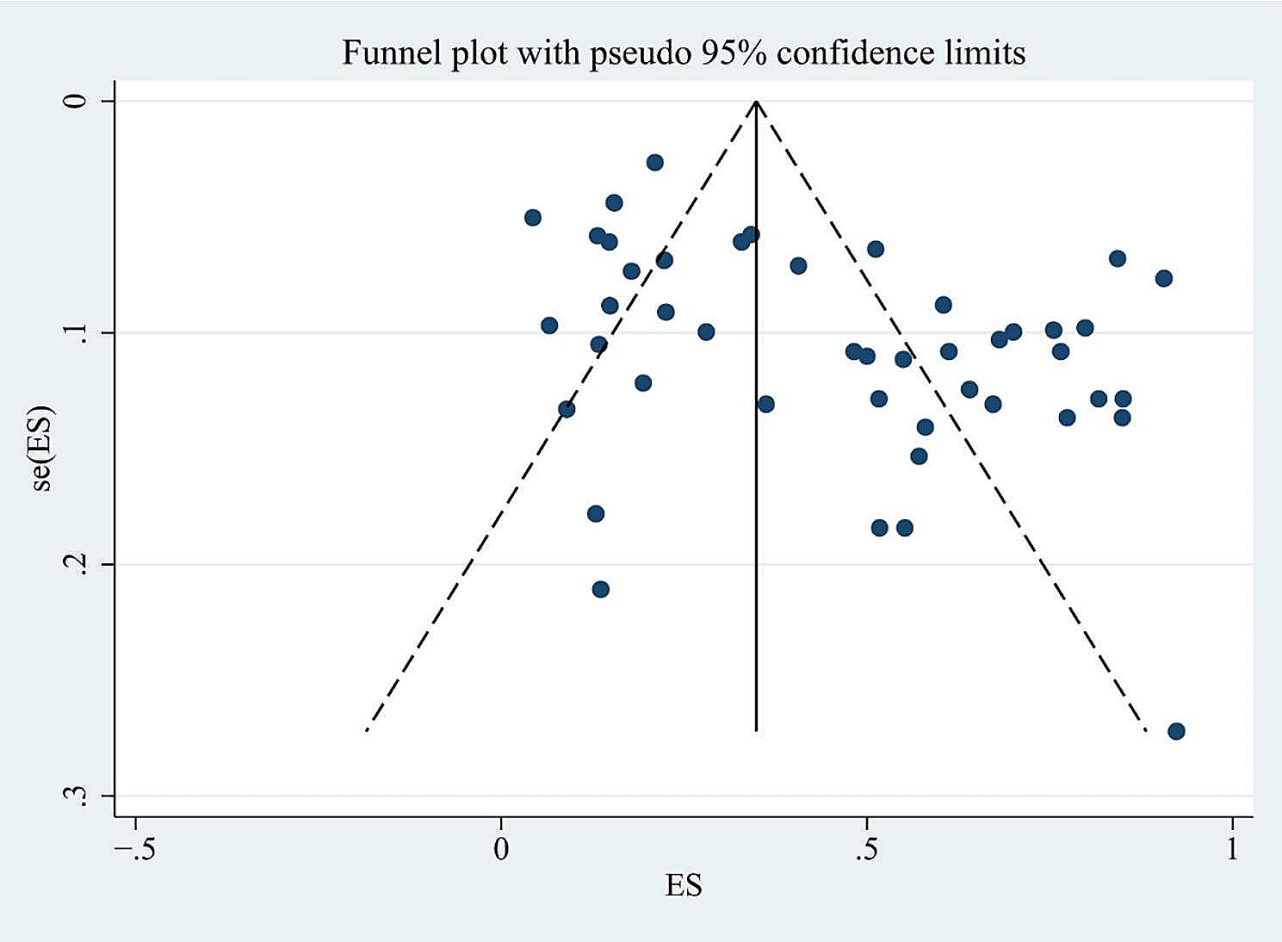
The funnel plot of vitamin D deficiency in children/adolescents with type 1 diabetes

For the whole sample of 6,995 individuals, the proportion of vitamin D deficiency in children and/or adolescents with T1D was 45% (95% CI; 37–54%; *P* < 0.01; Fig. 2). The analyses further indicated heterogeneity between studies (I-square [*I*^2^] = 97.94%, *P* < 0.001), and publication bias could be observed on the funnel plot. Publication bias in studies assessing the total proportion of vitamin D deficiency in T1D patients was analyzed using Begg’s test (z = 1.88; *P* = 0.060), Egger’s test (*P* = 0.000) and a funnel plot (Fig. 3).

Subgroup analyses were carried out according to the publication year, study design, classification of vitamin D, season and geographical region of the studies, with Table 2 presenting the estimated proportion of patients with vitamin D deficiency after the analysis.

**Table 2:** Summary of meta-analysis for the proportion of vitamin D deficiency in children/adolescents with T1D

Variable	Studies	Sample size	Cases	Vitamin D deficiency		
				95%CI	I^2^ (%)	*P*-value
**Total proportion**	45	6995	2436	0.45 (0.37, 0.54)	97.94	0.00
**Year**						
2009–2015	21	3921	1314	0.48 (0.36, 0.59)	97.91	0.00
2016–2022	24	3074	1122	0.43 (0.31, 0.56)	98.05	0.00
**Design**						
Cross-sectional	19	3233	841	0.31 (0.22, 0.40)	96.91	0.00
Case-control	23	2028	1129	0.58 (0.45, 0.72)	97.33	0.00
Cohort	1	244	125	0.51 (0.45, 0.58)	-	-
Longitudinal	2	1490	341	0.22 (0.20, 0.25)	-	-
**Geographical region**						
Africa	3	162	104	0.65 (0.42, 0.85)	-	-
Oceania	2	573	85	0.15 (0.12, 0.18)	-	-
Europe	11	1323	636	0.50 (0.32, 0.69)	97.82	0.00
North America	9	2899	629	0.24 (0.15, 0.34)	96.42	0.00
Asia	20	2038	982	0.54 (0.40, 0.68)	97.39	0.00
**Latitude**						
Low	3	307	217	0.50 (0.12, 0.88)	-	-
Mid-Low	13	1749	636	0.56 (0.38, 0.72)	97.86	0.00
Mid	27	4608	1445	0.39 (0.29, 0.50)	97.85	0.00
Mid-High	2	331	138	0.42 (0.37, 0.47)	-	-
**VD Classify**						
< 30ng/ml	2	199	170	0.87 (0.82, 0.92)	-	-
< 25ng/ml	1	104	83	0.80 (0.71, 0.87)	-	-
< 20ng/ml	29	3143	1394	0.49 (0.39, 0.60)	96.87	0.00
< 15ng/ml	6	1237	279	0.24 (0.11, 0.41)	97.31	0.00
< 12ng/ml	4	2099	386	0.14 (0.09, 0.20)	89.17	0.00
< 10ng/ml	3	213	124	0.67 (0.26, 0.97)	-	-
**Seasons**						
Winter	6	530	240	0.50 (0.37, 0.64)	85.06	0.00
Summer	6	530	99	0.17 (0.08, 0.27)	81.52	0.00
Spring	4	412	117	0.28 (0.23, 0.33)	4.19	0.37
Fall	4	412	74	0.20 (0.12, 0.29)	53.33	0.09

All the included studies were published between from 2009 to 2022. Twenty-one studies were published between 2009 and 2015, and 24 were published between 2016 and 2022. In contrast with the data from the previous six years (48%, 95% CI; 36–59%), more recent publications tended to yield a low proportion of vitamin D deficiency (43%, 95% CI; 31–56%). By comparing study designs, the subgroup analysis showed that a greater proportion of patients with vitamin D deficiency could be found in case‒control studies (58%, 95% CI; 45–72%), followed by one cohort study (51%, 95% CI; 45–58%) and 19 cross-sectional studies (31%, 95% CI; 22–40%), with the lowest proportion identified for 2 longitudinal studies (22%, 95% CI; 20–25%), but with significant heterogeneity. The proportion of vitamin D deficiency in children and/or adolescents with T1D was highest in Africa (65%, 95% CI; 42–85%), followed by Asia (54%, 95% CI; 40–68%), Europe (50%, 95% CI; 32–69%), North America (24%, 95% CI; 15–34%) and Oceania (15%, 95% CI; 12–18%), with significant differences among the five subgroups (*P* < 0.01). The proportion of vitamin D deficiency in children and/or adolescents with T1D at low-mid latitudes was 56% (95% CI; 38–72%), followed by that in children at low latitudes (50%, 95% CI; 12–88%), at mid-high latitudes (42%, 95% CI; 37–47%) and at middle latitudes (39%, 95% CI; 29–50%). A higher proportion of patients with a vitamin D deficiency was detected at 30 ng/ml (87%, 95% CI; 82–92%), followed by 25 ng/ml (80%, 95% CI; 71–87%), 10 ng/ml (67%, 95% CI; 26–97%), 20 ng/ml (49%, 95% CI; 39–60%), and 15 ng/ml (24%, 95% CI; 11–41%), with the lowest proportion identified at 12 ng/ml (14%, 95% CI; 9–20%). Subgroup analyses for different seasons showed that the proportion of individuals with vitamin D deficiency in winter tended to be significantly greater than that in summer (50%, 95% CI; 37–64% vs. 17%, 95% CI; 8–27%). In addition, studies conducted in spring reported a greater proportion of individuals with vitamin D deficiency (28%, 95% CI; 23–33%) than did those conducted in autumn (20%, 95% CI; 12–29%), but these differences were not significant (*P* > 0.01).

Sensitivity analysis was carried out to examine the influence of any particular study. To determine whether potential publication bias existed in the reviewed literature, Egger’s test was also carried out. The results of Egger’s test (*P* < 0.05) did suggest the existence of publication bias. Thus the publication bias of this study was corrected using the trim-and-fill method. The results showed that publication bias had little effect on the combined amount of results, indicating that the robustness of the results of this study was high.

Thirty-five studies involving 5,862 participants were included in the meta-analysis of the rate of vitamin D insufficiency among children and/or adolescents with T1D. In this case, the random effects model indicated that the cumulative proportion was 33.0% (95% CI; 27–38%). Considerable heterogeneity was also observed across studies *(I*^2^ = 94.27%, *P* < 0.01). Analyses of publication bias for studies estimating the total proportion of patients with vitamin D insufficiency were also conducted, with biases determined based on Begg’s test (z = 0.67; *P* = 0.504), Egger’s test (*P* = 0.614) and the funnel plot.

Thirty-nine studies, grouping 6,490 individuals from Europe (*n* = 11), Asia (*n* = 17), Africa (*n* = 1), North America (*n* = 9), and Oceania (*n* = 1), assessed the proportion of vitamin D sufficiency in children and/or adolescents with T1D. In this case, the proportion was estimated to be 27% (95% CI; 19–35%; *I*^2^ = 97.87%). Analyses of publication bias for studies estimating the total proportion of patients with sufficient vitamin D concentrations were also performed, with biases determined as before (i.e., with Begg’s test (z = 0.11; *P* = 0.913), Egger’s test (*P* = 0.007) and the funnel plot). Sensitivity analyses further revealed that 2 studies were off-center, and after omitting it [37, 64], the biases were again determined by both Begg’s test (z = 0.29; *P* = 0.773) and Egger’s test (*P* = 0.509).

## Discussion

This systematic review and meta-analysis comprehensively assessed the proportion of vitamin D deficiency in children and/or adolescents with T1D from a global perspective. The pooled estimate showed that vitamin D deficiency was prevalent among children and/or adolescents with T1D. As suggested by the present study, the rate of vitamin D deficiency in this particular group was high at 45%, which was high according to 45 studies involving 6,995 respondents. In addition, the proportions of patients with vitamin D insufficiency and vitamin D sufficiency were 33% and 27%, respectively. These findings may help to improve public health interventions for decreasing the proportion of vitamin D deficiency in children and/or adolescents with T1D. Moreover, these finding may serve as a reminder that greater attention should be given to vitamin D deficiency in clinical practice.

The high proportion of vitamin D deficiency in children and/or adolescents with T1D may be explained by the fact that vitamin D is lipophilic and is mainly absorbed in the small intestine before further processing in the skin, liver and kidneys to the biologically active compound 1,25-dihydroxyvitamin D. In addition, the absorption of lipophilic substances is dependent on a variety of intricate processes that require an intact epithelium in the small intestine but also on extraintestinal factors, such as the release of lipase from the pancreas and bile from the liver [66].

High heterogeneity was identified across the included studies. Subgroup analysis further revealed marked between-study variability in estimates of the proportion of patients with vitamin D deficiency. For instance, the results of subgroup analysis by publication year showed that more recent publications tended to yield low vitamin D deficiency proportion estimates. This discrepancy might be due to increasing awareness of the importance of vitamin D supplements and sun exposure. Furthermore, by comparing study designs, the present study revealed that the proportion of patients with vitamin D deficiency in case‒control studies tended to be greater than that in other studies. This inconsistency clearly indicated that different study designs could yield different estimates of the proportion of patients with vitamin D deficiency.

The other study-specific factor that we considered in the subgroup analysis was geographical region. Compared to those in other regions, we found that the proportion of vitamin D deficiency in children and/or adolescents with T1D in Africa tended to be greater than that in Asia (65% vs. 54%), followed by Europe (50%), North America (24%) and Oceania (15%), thus indicating that geographical regions could partly explain some of the variance. This could have been due to differences in culture, religion, ethnicity, dietary habits and forms of exercise. Indeed, low vitamin D levels in some populations are related to social customs such as the avoidance of sunlight or even breastfeeding without any vitamin D supplementation [67]. Due to differences in study design, only one study [16] statistically assessed dietary fortification as an influencing factor among the included studies, which is also one of the underlying reasons for the bias. Another important aspect to consider is that the recommended vitamin D intake for children and adolescents varies by country. For instance, the American Academy of Pediatrics recommends a minimum daily intake of 200 U/d of vitamin D beginning in the first 2 months after birth and continuing through adolescence [68]. In China, vitamin D supplementation is recommended to begin within a few days after birth, and at least 400 U/d is recommended during infancy to adolescence. Daily oral vitamin D supplementation is recommended. When compliance is poor, large doses of vitamin D can be administered orally. When gastrointestinal disease occurs, large doses of vitamin D can be administered intramuscularly [69]. According to global consensus recommendations on the prevention and management of nutritional rickets, at more than 12 months of age, all children need to meet their nutritional requirement for vitamin D through diet and/or supplementation, which is at least 600 U/d [70]. In addition to the fact that individuals originated from different territorial areas, participant characteristics such as age and ethnicity also varied among studies. Some participants could also have had higher vitamin D requirements for bone growth, especially during pubertal growth spurts [71], further contributing to the heterogeneity.

According to our subgroup analysis, one of the most important factors was the cutoff value for vitamin D deficiency. Compared with a cutoff value of < 25 ng/ml, a cutoff value of < 30 ng/ml was associated with a significantly greater incidence of vitamin D deficiency. This procedure was followed by a cutoff value of < 10 ng/ml, a cutoff value of < 20 ng/ml, and a cutoff value of < 15 ng/ml, with the lowest proportion identified for a cutoff value of < 12 ng/ml. This may be due to the small sample size. This variability could be partly attributed to the lack of standardized 25(OH)D measurements in vitamin D research. Beyond that, within a given methodology, there are several possible causes for differences, such as lot-to-lot variation in manufacturer reagents or differences in subjects included in different studies.

Subgroup analysis also revealed an interesting findings. The present study revealed that the proportion of vitamin D deficiency in children and/or adolescents with T1D in winter tended to be significantly greater than that in summer. In addition, these findings add weight to the conclusion that the proportion of vitamin D deficiency in children and/or adolescents with T1D at mid-low latitudes tends to be greater than that at low latitudes (56% vs. 50%), followed by at mid- to high latitudes (42%) and finally at middle latitudes (39%). This discrepancy might be because there is a longer sunlight duration in summer than in winter. While separating research into subgroups revealed numerous noteworthy differences, post hoc comparisons should be interpreted with caution. The heterogeneity in proportions between studies was not satisfactorily explained by any of the parameters examined, with *I*^2^ values being greater than 65% for all subgroups.

The current research has some limitations. First, all the studies were clinic- or hospital-based, which could have affected the true prevalence in the general population. Second, the selected studies included cross-sectional, case‒control, cohort and longitudinal studies that were limited by study design and therefore had an inevitable risk of bias. Third, there is currently no internationally agreed upon classification standard for vitamin D deficiency, and as such, there may be significant variations during reporting. Finally, the possibility of publication bias could not be fully excluded by Egger’s test. Trim and fill analysis was also conducted, and the results did not change the estimate, indicating that the results are robust to the possibility of unpublished studies.

Vitamin D may have direct effects on β cells, including improving insulin secretion, enhancing the expression of the vitamin D receptor and improving islet morphology [72]. As vitamin D intake is a potentially important and modifiable behavioral target, clinical professionals need to screen for vitamin D deficiency in children and/or adolescents with T1D to guide appropriate supplementation.

## Conclusion

This review demonstrated that vitamin D deficiency affects 45% of children and/or adolescents with T1D, and children and/or adolescents with T1D in winter had an increased susceptibility to vitamin D deficiency compared with those in other seasons. These results contribute to a better understanding of vitamin D deficiency in children and/or adolescents with T1D and demonstrate the importance of assessing vitamin D deficiency in children and/or adolescents with diabetes. Preventive strategies and interventions to address vitamin D deficiency in children and/or adolescents with T1D should be considered in healthcare settings. Future research should focus on increasing our understanding of the temporal relationship between diabetes and vitamin D deficiency.

### Electronic supplementary material

Below is the link to the electronic supplementary material.

Supplementary Material 1

Supplementary Material 2

## Data Availability

The datasets used and/or analysed during the current study are available from the corresponding/first author on reasonable request.

## Abbreviations

T1D: Type 1 diabetes
DKA: Diabetic Ketoacidosis
CI: Confidence Interval
VD: vitamin D
PRISMA: The Preferred Reporting Items for Systematic Reviews and Meta-Analyses
MeSH: Medical Subject Headings
NOS: The Newcastle–Ottawa Scale
AHRQ: The Agency for Healthcare Research and Quality
WHO: The World Health Organization
ADA: The American Diabetes Association
EURODIAB: The European Diabetes collaboration
HPLC: High-Performance Liquid Chromatography
