# Supplementary material for: Proportion of vitamin D deficiency in children/adolescents with type 1 diabetes: a systematic review and meta-analysis
Source: BMC Pediatr. 2024 Mar 16;24:192. doi: 10.1186/s12887-024-04683-5 (PMC10943883; doi:10.1186/s12887-024-04683-5)
Supplement: Supplementary file 1 — Supplementary Material 1 [file 12887_2024_4683_MOESM1_ESM.doc]

**Appendix S1. The detailed search strategy.**

Studies published from the inception of the database up to the end of April 2022

1. **The Cochrane Library**

| (Diabetes Mellitus, Insulin-Dependent):ab,ti,kw OR (Diabetes Mellitus, Insulin Dependent):ab,ti,kw OR (Insulin-Dependent Diabetes Mellitus):ab,ti,kw OR (Diabetes Mellitus, Juvenile-Onset):ab,ti,kw OR (Diabetes Mellitus, Juvenile Onset):ab,ti,kw OR (Juvenile-Onset Diabetes Mellitus):ab,ti,kw OR (IDDM):ab,ti,kw OR (Juvenile-Onset Diabetes):ab,ti,kw OR (Diabetes, Juvenile-Onset):ab,ti,kw OR (Juvenile Onset Diabetes):ab,ti,kw OR (Diabetes Mellitus, Sudden-Onset):ab,ti,kw OR (Diabetes Mellitus, Sudden Onset):ab,ti,kw OR (Sudden-Onset Diabetes Mellitus):ab,ti,kw OR (Type 1 Diabetes Mellitus):ab,ti,kw OR (Diabetes Mellitus, Insulin-Dependent, 1):ab,ti,kw OR (Insulin-Dependent Diabetes Mellitus 1):ab,ti,kw OR (Insulin Dependent Diabetes Mellitus 1):ab,ti,kw OR (Type 1 Diabetes):ab,ti,kw OR (Diabetes, Type 1):ab,ti,kw OR (Diabetes Mellitus, Type I):ab,ti,kw OR (Diabetes, Autoimmune):ab,ti,kw OR (Autoimmune Diabetes):ab,ti,kw |
| --- |
| (Adolescents):ab,ti,kw OR (Adolescence):ab,ti,kw OR (Teens):ab,ti,kw OR (Teen):ab,ti,kw OR (Teenagers):ab,ti,kw OR (Teenager):ab,ti,kw OR (Youth):ab,ti,kw OR (Youths):ab,ti,kw OR (Adolescents, Female):ab,ti,kw OR (Adolescent, Female):ab,ti,kw OR (Female Adolescent):ab,ti,kw OR (Female Adolescents):ab,ti,kw OR (Adolescents, Male):ab,ti,kw OR (Adolescent, Male):ab,ti,kw OR (Male Adolescent):ab,ti,kw OR (Male Adolescents):ab,ti,kw OR (Child):ab,ti,kw OR (Children):ab,ti,kw OR (Adolescent):ab,ti,kw |
| (25-hydroxyvitamin D):ab,ti,kw OR (Vitamin D):ab,ti,kw OR (25-hydroxyvitamin D):ab,ti,kw OR (Vitamin D):ab,ti,kw |

1. **Web of Science**

| Diabetes Mellitus, Insulin-Dependent OR Diabetes Mellitus, Insulin Dependent OR Insulin-Dependent Diabetes Mellitus OR Diabetes Mellitus, Juvenile-Onset OR Diabetes Mellitus, Juvenile Onset OR Juvenile-Onset Diabetes Mellitus OR IDDM OR Juvenile-Onset Diabetes OR Diabetes, Juvenile-Onset OR Juvenile Onset Diabetes OR Diabetes Mellitus, Sudden-Onset OR Diabetes Mellitus, Sudden Onset OR Sudden-Onset Diabetes Mellitus OR Type 1 Diabetes Mellitus OR Diabetes Mellitus, Insulin-Dependent, 1 OR Insulin-Dependent Diabetes Mellitus 1 OR Insulin Dependent Diabetes Mellitus 1 OR Type 1 Diabetes OR Diabetes, Type 1 OR Diabetes Mellitus, Type I OR Diabetes, Autoimmune OR Autoimmune Diabetes |
| --- |
| Adolescents OR Adolescence OR Teens OR Teen OR Teenagers OR Teenager OR Youth OR Youths OR Adolescents, Female OR Adolescent, Female OR Female Adolescent OR Female Adolescents OR Adolescents, Male OR Adolescent, Male OR Male Adolescent OR Male Adolescents OR Child OR Children OR Adolescent |
| 25-hydroxyvitamin D OR Vitamin D OR 25-hydroxyvitamin D OR Vitamin D |

**(3)Ovid_Medline**

| Diabetes Mellitus, Insulin-Dependent or Diabetes Mellitus, Insulin Dependent or Insulin-Dependent Diabetes Mellitus or Diabetes Mellitus, Juvenile-Onset or Diabetes Mellitus, Juvenile Onset or Juvenile-Onset Diabetes Mellitus or IDDM or Juvenile-Onset Diabetes or Diabetes, Juvenile-Onset or Juvenile Onset Diabetes or Diabetes Mellitus, Sudden-Onset or Diabetes Mellitus, Sudden Onset or Sudden-Onset Diabetes Mellitus or Type 1 Diabetes Mellitus or Diabetes Mellitus, Insulin-Dependent, 1 or Insulin-Dependent Diabetes Mellitus 1 or Insulin Dependent Diabetes Mellitus 1 or Type 1 Diabetes or Diabetes, Type 1 or Diabetes Mellitus, Type I or Diabetes, Autoimmune or Autoimmune Diabetes |
| --- |
| Adolescents or Adolescence or Teens or Teen or Teenagers or Teenager or Youth or Youths or Adolescents, Female or Adolescent, Female or Female Adolescent or Female Adolescents or Adolescents, Male or Adolescent, Male or Male Adolescent or Male Adolescents or Child or Children or Adolescent |
| 25-hydroxyvitamin D OR Vitamin D OR 25-hydroxyvitamin D OR Vitamin D |

**(4)ProQuest**

| "Diabetes Mellitus, Type 1" OR "Diabetes Mellitus, Insulin-Dependent" OR "Diabetes Mellitus, Insulin Dependent" OR "Insulin-Dependent Diabetes Mellitus" OR "Diabetes Mellitus, Juvenile-Onset" OR "Diabetes Mellitus, Juvenile Onset" OR "Juvenile-Onset Diabetes Mellitus" OR "IDDM" OR "Juvenile-Onset Diabetes" OR "Diabetes, Juvenile-Onset" OR "Juvenile Onset Diabetes" OR "Diabetes Mellitus, Sudden-Onset" OR "Diabetes Mellitus, Sudden Onset" OR "Sudden-Onset Diabetes Mellitus" OR "Type 1 Diabetes Mellitus" OR "Diabetes Mellitus, Insulin-Dependent, 1" OR "Insulin-Dependent Diabetes Mellitus 1" OR "Insulin Dependent Diabetes Mellitus 1" OR "Type 1 Diabetes" OR "Diabetes, Type 1" OR "Diabetes Mellitus, Type I" OR "Diabetes, Autoimmune" OR "Autoimmune Diabetes" |
| --- |
| "Adolescents" OR "Adolescence" OR "Teens" OR "Teen" OR "Teenagers" OR "Teenager" OR "Youth" OR "Youths" OR "Adolescents, Female" OR "Adolescent, Female" OR "Female Adolescent" OR "Female Adolescents" OR "Adolescents, Male" OR "Adolescent, Male" OR "Male Adolescent" OR "Male Adolescents" OR "Child" OR "Children" OR "Adolescent" |
| "25-hydroxyvitamin D" OR "Vitamin D" OR "25-hydroxyvitamin D" OR "Vitamin D" |

**(5)Embase**

| 'Diabetes Mellitus, Type 1':ab,ti OR 'Diabetes Mellitus, Insulin-Dependent':ab,ti OR 'Diabetes Mellitus, Insulin Dependent':ab,ti OR 'Insulin-Dependent Diabetes Mellitus':ab,ti OR 'Diabetes Mellitus, Juvenile-Onset':ab,ti OR 'Diabetes Mellitus, Juvenile Onset':ab,ti OR 'Juvenile-Onset Diabetes Mellitus':ab,ti OR 'IDDM':ab,ti OR 'Juvenile-Onset Diabetes':ab,ti OR 'Diabetes, Juvenile-Onset':ab,ti OR 'Juvenile Onset Diabetes':ab,ti OR 'Diabetes Mellitus, Sudden-Onset':ab,ti OR 'Diabetes Mellitus, Sudden Onset':ab,ti OR 'Sudden-Onset Diabetes Mellitus':ab,ti OR 'Type 1 Diabetes Mellitus':ab,ti OR 'Diabetes Mellitus, Insulin-Dependent, 1':ab,ti OR 'Insulin-Dependent Diabetes Mellitus 1':ab,ti OR 'Insulin Dependent Diabetes Mellitus 1':ab,ti OR 'Type 1 Diabetes':ab,ti OR 'Diabetes, Type 1':ab,ti OR 'Diabetes Mellitus, Type I':ab,ti OR 'Diabetes, Autoimmune':ab,ti OR 'Autoimmune Diabetes':ab,ti |
| --- |
| 'Adolescents':ab,ti OR 'Adolescence':ab,ti OR 'Teens':ab,ti OR 'Teen':ab,ti OR 'Teenagers':ab,ti OR 'Teenager':ab,ti OR 'Youth':ab,ti OR 'Youths':ab,ti OR 'Adolescents, Female':ab,ti OR 'Adolescent, Female':ab,ti OR 'Female Adolescent':ab,ti OR 'Female Adolescents':ab,ti OR 'Adolescents, Male':ab,ti OR 'Adolescent, Male':ab,ti OR 'Male Adolescent':ab,ti OR 'Male Adolescents':ab,ti OR 'Child':ab,ti OR 'Children':ab,ti OR 'Adolescent':ab,ti |
| '25-hydroxyvitamin D':ab,ti OR 'Vitamin D':ab,ti OR '25-hydroxyvitamin D':ab,ti OR 'Vitamin D' |
