# Supplementary material for: Proportion of vitamin D deficiency in children/adolescents with type 1 diabetes: a systematic review and meta-analysis
Source: BMC Pediatr. 2024 Mar 16;24:192. doi: 10.1186/s12887-024-04683-5 (PMC10943883; doi:10.1186/s12887-024-04683-5)
Supplement: Supplementary file 2 — Supplementary Material 2 [file 12887_2024_4683_MOESM2_ESM.doc]

**Appendix S2 Quality assessment**

1. The Newcastle–Ottawa Scale (NOS)

| Design | Auther | Is the case definition adequate? | | Representativeness of the cases | Selection of controls | | Definition of controls | Comparability of cases and controls on the basis of the design or analysis | Ascertainment of exposure | Same method of ascertainment for cases and controls | Non-Response rate | Score |
| --- | --- | --- | --- | --- | --- | --- | --- | --- | --- | --- | --- | --- |
| Case-control | Bener 2009 | Venous blood glucose values equal or>6.7mmol/l | ❤ | ❤ | Healthy subjects | ❤ | ❤ | ❤❤ | ❤ | ❤ | ❤ | 9 |
| Case-control | Borkar 2010 | ADA | ❤ | ❤ | Healthy subjects | ❤ | ❤ | ❤❤ | ❤ | ❤ | ❤ | 9 |
| Case-control | Daga 2012 | Not stated |  | ❤ | Healthy subjects | ❤ | ❤ | ❤❤ | ❤ | ❤ | ❤ | 8 |
| Case-control | Azab 2013 | WHO | ❤ | ❤ | Healthy subjects | ❤ | ❤ | ❤❤ | ❤ | ❤ | ❤ | 9 |
| Case-control | Lieberman 2013 | Islet cell antibody | ❤ | ❤ | Healthy subjects | ❤ | ❤ | ❤❤ | ❤ | ❤ | ❤ | 9 |
| Case-control | Greer 2013 | Not stated |  | ❤ | Children attending the Ear, Nose and Throat Clinic |  | ❤ | ❤❤ | ❤ | ❤ | ❤ | 8 |
| Case-control | Franchi 2013 | ADA | ❤ | ❤ | The children admitted in the same period for other reasons |  | ❤ | ❤❤ | ❤ | ❤ | ❤ | 8 |
| Case-control | Jung 2014 | Not stated |  | ❤ | Healthy subjects | ❤ | ❤ | ❤❤ | ❤ | ❤ | ❤ | 8 |
| Case-control | Wierzbicka 2016 | Not stated |  | ❤ | Healthy subjects | ❤ | ❤ | ❤❤ | ❤ | ❤ | ❤ | 8 |
| Case-control | Rasoul 2016 | ISPAD/WHO | ❤ | ❤ | Healthy subjects | ❤ | ❤ | ❤❤ | ❤ | ❤ | ❤ | 9 |
| Case-control | Kim H 2017 | Not stated |  | ❤ | Healthy subjects | ❤ | ❤ | ❤❤ | ❤ | ❤ | ❤ | 8 |
| Design | Auther | Is the case definition adequate? | | Representativeness of the cases | Selection of controls | | Definition of controls | Comparability of cases and controls on the basis of the design or analysis | Ascertainment of exposure | Same method of ascertainment for cases and controls | Non-Response rate | Score |
| Case-control | Ziaei-Kajbaf 2018 | Not stated |  | ❤ | The outpatient clinic |  | ❤ | ❤❤ | ❤ | ❤ | ❤ | 7 |
| Case-control | Liu 2018 | Not stated |  | ❤ | The outpatient clinic |  | ❤ | ❤❤ | ❤ | ❤ | ❤ | 7 |
| Case-control | Federico 2018 | Not stated |  | ❤ | Non-diabetic control |  | ❤ | ❤❤ | ❤ | ❤ | ❤ | 7 |
| Case-control | Bae 2018 | Not stated |  | ❤ | Healthy subjects | ❤ | ❤ | ❤❤ | ❤ | ❤ | ❤ | 8 |
| Case-control | Sonia 2016 | ADSC | ❤ | ❤ | Emergency or admitted to hospital in the same period |  | ❤ | ❤❤ | ❤ | ❤ | ❤ | 8 |
| Case-control | Mansi 2021 | Not stated |  | ❤ | Non-diabetic control |  | ❤ | ❤❤ | ❤ | ❤ | ❤ | 7 |
| Case-control | Soliman 2015 | ADA | ❤ | ❤ | Healthy subjects | ❤ | ❤ | ❤❤ | ❤ | ❤ | ❤ | 9 |
| Case-control | Rochmah 2022 | ISPAD | ❤ | ❤ | Healthy subjects | ❤ | Not stated | ❤❤ | ❤ | ❤ | ❤ | 8 |
| Case-control | Setty-Shah2014 | ADA | ❤ | ❤ | Healthy subjects | ❤ | ❤ | ❤❤ | ❤ | ❤ | ❤ | 9 |
| Case-control | Ghandchi 2012 | Not stated |  | ❤ | Healthy subjects | ❤ | ❤ | ❤❤ | ❤ | ❤ | ❤ | 8 |
| Case-control | Biliaieva 2022 | Not stated |  | ❤ | Healthy subjects | ❤ | ❤ | Not stated | ❤ | ❤ | ❤ | 6 |
| Case-control | Polat 2022 | Not stated |  | ❤ | Psychiatric patients |  | ❤ | ❤❤ | ❤ | ❤ | ❤ | 7 |

| **Design** | **Auther** | Representativeness of the exposed cohort | | Selection of the non exposed cohort | Ascertainment of exposure | Demonstration that outcome of interest was not present at start of study | Comparability of cohorts on the basis of the design or analysis | Assessment of outcome | Was follow-up long enough for outcomes to occur | Adequacy of follow up of cohorts | score |
| --- | --- | --- | --- | --- | --- | --- | --- | --- | --- | --- | --- |
| Cohort | Raab 2014 | ADA | ❤ | ❤ | ❤ | ❤ | ❤❤ | ❤ | ❤ | ❤ | 9 |

1. **The Agency for Healthcare Research and Quality (AHRQ)**

| **AHRQ** | Janner 2010 | Svoren 2009 | Mutlu 2011 | Thnc 2011 | Vojtkova 2012 | Ataie-Jafari 2012 | Al-Zubeidi 2016 | Al 2016 | Zambrana-Calví 2016 | Al Sawah 2016 | Giri 2017 | ALkharashi 2019 | Segovia-Ortí 2020 | Carakushansky 2020 | Saki 2017 | Yeshayahu 2012 | Zabeen 2021 | KOR 2018 | The 2013 | Savastio 2016 | Kaur 2011 |
| --- | --- | --- | --- | --- | --- | --- | --- | --- | --- | --- | --- | --- | --- | --- | --- | --- | --- | --- | --- | --- | --- |
| 1) Define the source of information (survey, record review) | 1 | 1 | 1 | 0 | 1 | 1 | 1 | 1 | 1 | 1 | 1 | 1 | 1 | 1 | 1 | 1 | 1 | 1 | 1 | 1 | 1 |
| 2) List inclusion and exclusion criteria for exposed and unexposed subjects (cases and controls) or refer to previous publications | 1 | 1 | 1 | 1 | 1 | 1 | 1 | 1 | 1 | 1 | 1 | 1 | 1 | 1 | 1 | 1 | 1 | 0 | 1 | 1 | 0 |
| 3) Indicate time period used for identifying patients | 1 | 1 | 1 | 0 | 1 | 1 | 1 | 1 | 1 | 1 | 0 | 1 | 1 | 1 | 1 | 1 | 1 | 1 | 1 | 1 | 1 |
| **AHRQ** | Janner 2010 | Svoren 2009 | Mutlu 2011 | Thnc 2011 | Vojtkova 2012 | Ataie-Jafari 2012 | Al-Zubeidi 2016 | Al 2016 | Zambrana-Calví 2016 | Al Sawah 2016 | Giri 2017 | ALkharashi 2019 | Segovia-Ortí 2020 | Carakushansky 2020 | Saki 2017 | Yeshayahu 2012 | Zabeen 2021 | KOR 2018 | The 2013 | Savastio 2016 | Kaur 2011 |
| 4) Indicate whether or not subjects were consecutive if not population-based | 1 | 1 | 1 | 0 | 1 | 0 | 1 | 1 | 1 | 1 | 0 | 1 | 1 | 1 | 1 | 1 | 1 | 1 | 1 | 1 | 1 |
| 5) Indicate if evaluators of subjective components of study were masked to other aspects of the status of the participants | 1 | 1 | 1 | 1 | 1 | 1 | 1 | 1 | 1 | 1 | 1 | 1 | 1 | 1 | 1 | 1 | 1 | 0 | 1 | 1 | 0 |
| 6) Describe any assessments undertaken for quality assurance purposes (e.g., test/retest of primary outcome measurements) | 1 | 1 | 1 | 1 | 1 | 1 | 1 | 1 | 1 | 1 | 1 | 1 | 1 | 1 | 1 | 1 | 1 | 0 | 1 | 1 | 1 |
| 7) Explain any patient exclusions from analysis | 1 | 1 | 1 | 1 | 1 | 1 | 1 | 1 | 1 | 1 | 1 | 1 | 0 | 1 | 1 | 1 | 1 | 0 | 1 | 1 | 0 |
| 8) Describe how confounding was assessed and/or controlled. | 0 | 0 | 0 | 0 | 0 | 0 | 0 | 1 | 0 | 0 | 0 | 0 | 0 | 1 | 0 | 0 | 0 | 0 | 1 | 0 | 0 |
| 9) If applicable, explain how missing data were handled in the analysis | 1 | 1 | 1 | 1 | 1 | 1 | 1 | 1 | 1 | 1 | 1 | 1 | 1 | 1 | 1 | 1 | 1 | 1 | 1 | 1 | 0 |
| **AHRQ** | Janner 2010 | Svoren 2009 | Mutlu 2011 | Thnc 2011 | Vojtkova 2012 | Ataie-Jafari 2012 | Al-Zubeidi 2016 | Al 2016 | Zambrana-Calví 2016 | Al Sawah 2016 | Giri 2017 | ALkharashi 2019 | Segovia-Ortí 2020 | Carakushansky 2020 | Saki 2017 | Yeshayahu 2012 | Zabeen 2021 | KOR 2018 | The 2013 | Savastio 2016 | Kaur 2011 |
| 10) Summarize patient response rates and completeness of data collection | 1 | 1 | 1 | 1 | 1 | 1 | 1 | 1 | 1 | 1 | 1 | 1 | 1 | 1 | 1 | 1 | 1 | 1 | 1 | 1 | 1 |
| 11) Clarify what follow-up, if any, was expected and the percentage of patients for which incomplete data or follow-up was obtained | 1 | 1 | 1 | 1 | 1 | 1 | 1 | 1 | 1 | 0 | 1 | 1 | 1 | 1 | 1 | 1 | 1 | 0 | 1 | 1 | 0 |
| **score** | 10 | 10 | 10 | 7 | 10 | 9 | 10 | 11 | 10 | 9 | 8 | 10 | 9 | 11 | 10 | 10 | 10 | 5 | 11 | 10 | 5 |
